# Cingulate activity and fronto-temporal connectivity in people with prodromal signs of psychosis

**DOI:** 10.1016/j.neuroimage.2009.08.038

**Published:** 2010-01-01

**Authors:** Paul Allen, Klaas E. Stephan, Andrea Mechelli, Fern Day, Nicholas Ward, Jeffery Dalton, Steven C. Williams, Philip McGuire

**Affiliations:** a Division of Psychological Medicine, Institute of Psychiatry, King's College London, UK; b Neuroimaging Research Group, Institute of Psychiatry, King's College London, UK; c Laboratory for Social and Neural Systems Research, Institute for Empirical Research in Economics, University of Zurich, Switzerland; d Wellcome Trust Centre for Neuroimaging, Institute of Neurology, University College London, London, UK

**Keywords:** Frontotemporal connectivity, Prodromal psychosis, Anterior cingulate cortex, Dynamic causal modelling, DCM, Bayesian model selection, Magnetic Resonance Imaging

## Abstract

Schizophrenia is associated with fronto-temporal dysconnectivity, but it is not clear whether this is a risk factor for the disorder or is a consequence of the established illness. The aim of the present study was to use fMRI to investigate fronto-temporal connectivity in subjects with prodromal signs of schizophrenia using the Hayling Sentence Completion Task (HSCT). Thirty participants, 15 with an at risk mental state (ARMS) and 15 healthy controls were scanned whilst completing 80 sentence stems. The congruency and constraint of sentences varied across trials. Dynamic causal modelling (DCM) and Bayesian model selection (BMS) were used to compare alternative models of connectivity in a task related network. During the HSCT ARMS subjects did not differ from Healthy Controls in terms of fronto-temporal activation, i.e. there was neither a main effect of group nor a group-by-task interaction. However, there was both a significant main effect of group and a significant interaction in the anterior cingulate cortex (ACC), with greater ACC activity in the ARMS subjects. A systematic BMS procedure among 14 alternative DCMs including the ACC, middle frontal, and middle temporal gyri revealed intact task-dependent modulation of fronto-temporal effective connectivity in the ARMS group. However, ARMS subjects showed increased endogenous connection strength between the ACC and the middle temporal gyrus relative to healthy controls. Although task related fronto-temporal integration in the ARMS was intact, this may depend on increased engagement of the ACC which was not observed in healthy control subjects.

## Introduction

Disordered brain connectivity is thought to be a central pathophysiological feature of schizophrenia (Walterfang et al., 2006; Stephan et al., 2009a, 2009b). Recently a number of functional imaging (Boksman et al., 2005; Ragland et al., 2001; Wolf et al., 2007; Yoon et al., 2008) and diffusion tensor imaging (DTI) studies (Hubl et al., 2004; Rosenberger et al., 2008; Shergill et al., 2007) have provided data consistent with this view. In particular, the disconnection hypothesis of schizophrenia was motivated by initial positron emission tomography (PET) studies showing abnormal patterns of functional connectivity between prefrontal and temporal lobe regions (Friston, 1998; Friston and Frith, 1995; Frith et al., 1995). Subsequent experimental evidence, using functional Magnetic Resonance Imaging (fMRI), has been largely consistent with this hypothesis, reporting altered fronto-temporal connectivity in schizophrenia patients relative to healthy control subjects (Fletcher et al., 1999; Frith et al., 1995; Lawrie et al., 2002; Winder et al., 2007). It is not clear, however, whether dysconnectivity contributes to the development of schizophrenia or whether it is a consequence of the illness or its treatment (Konrad and Winterer, 2008). A recent review of structural neuroimaging and electrophysiology studies in first episode schizophrenia concluded that there is evidence that connectivity is altered in the early stage of the disorder (Begre and Koenig, 2008). Other studies using electroencephalography (Winterer et al., 2003) and DTI (Konrad et al., 2009) suggest that fronto-temporal connectivity is perturbed in subjects at increased genetic risk of schizophrenia. Using functional magnetic resonance imaging (fMRI), Whalley et al. (2005) studied the relatives of patients with the Hayling Sentence Completion Task (HSCT; (Burgess and Shallice, 1996). They found functional dysconnectivity between cerebral cortical, thalamic, and cerebellar regions, but no evidence of fronto-temporal dysconnectivity. They suggested that the latter might be a feature of established schizophrenia but not of groups at high genetic risk of the disorder.

Another approach is to study people at high risk as judged by clinical symptoms. Individuals with prodromal symptoms and signs of psychosis, such as attenuated psychotic symptoms and a decline in social and occupational function, are said to have an at risk mental state (ARMS), as these features are associated with a high risk of psychosis (Hafner, 2002; Yung et al., 1998). The ARMS is associated with abnormalities of regional brain function and structure that are often qualitatively similar to those seen in schizophrenia, but less marked (Fusar-Poli et al., 2007). Volumetric MRI studies have identified reductions in grey matter volume in prefrontal and temporal regions (Borgwardt et al., 2007; Meisenzahl et al., 2008; Pantelis et al., 2003) and fMRI studies have reported altered activation in frontal regions during tasks of executive functions and working memory (Broome et al., 2009; Morey et al., 2005). The aim of the present study was to use fMRI to investigate fronto-temporal connectivity in the ARMS. We used the HSCT, which requires participants to complete a sentence with a semantically related congruent word (response Initiation) or an unrelated incongruent word (response Suppression). We selected this task for two reasons. Firstly, performance of the HSCT is normally associated with robust engagement of the prefrontal and lateral temporal cortex. Secondly, we have recently shown that when healthy controls perform the HSCT effective connectivity between frontal and temporal regions is greater during response Suppression relative to Initiation (Allen et al., 2008). In the present study we used dynamic causal modelling (DCM) (Friston et al., 2003), a recently developed method to infer effective connectivity and its modulation by specific experimental contexts (e.g. task demands) from fMRI measurements.

We first tested the hypothesis that during the HSCT, ARMS subjects would show an altered pattern of frontal and temporal activation relative to healthy controls particularly during response Suppression, the most cognitively demanding of the task conditions. Using DCM, we then tested the hypothesis that task-dependent effective connectivity between frontal and temporal regions would be diminished in ARMS subjects compared to healthy controls.

## Materials and methods

### Participants

Thirty subjects (15 healthy controls and 15 with an ARMS) participated in the study. Results for the healthy controls have been previously reported by us (Allen et al. 2008). All were right-handed, spoke English as their first language, and had no history of neurological illness, drug, or alcohol dependence. The study had National Health Service UK Research Ethics Committee (CoREC) approval and all participants gave informed consent. FMRI data from both healthy controls and ARMS subjects were collected over the same time period. All participants had an estimated premorbid IQ in the normal range as assessed using the Wide Range Achievement Test—Revised (WRAT) (Jastak and Wilkinson, 1984). Exclusion criteria were a history of past or present psychiatric illness, significant head trauma or any CNS disease, current medical illness, and use of any regular medication in the last 2 months. No subjects had a significant history of drug or alcohol use. Any participants reporting excessive use of alcohol or recent recreational drug use (use of cannabis, stimulants, hallucinogens, or opiates in the 2 weeks prior to the fMRI scan) were also excluded.

#### Healthy controls

Fifteen healthy, right-handed male (*n* = 8) and female volunteers (*n* = 7) were recruited from the same geographical area as the ARMS group via advertisements and matched to the ARMS group in terms of age, years of education, and premorbid IQ (Table 1). Their self-reported ethnicity was white British (*n* = 10), black (*n* = 3), and mixed (*n* = 2).

#### Subjects with an At Risk Mental State (ARMS)

Fifteen right-handed males (*n* = 9) and females (*n* = 6) participated. Mean age, years of education, and estimated premorbid IQ are shown in Table 1. Their self-reported ethnicity was white British (*n* = 9), black (*n* = 2), and mixed (*n* = 4). ARMS subjects were recruited via OASIS (Outreach and Support in South London), a clinical service for people at high risk of developing psychosis (Broome et al., 2005). The ARMS was defined according to the Personal Assessment and Crisis Evaluation (PACE) criteria (Yung et al., 1998) and the diagnosis was made via a detailed clinical assessment using the Comprehensive Assessment of At Risk Mental States (Phillips et al., 2000). Subjects met one or more of the following criteria, namely, a) attenuated psychotic symptoms, b) brief limited intermittent psychosis, or c) a recent decline in function, together with either schizotypal personality disorder or a first degree relative with a psychotic disorder. All ARMS subjects were experiencing attenuated psychotic symptoms, four had also experienced a brief limited intermittent psychosis, and three had a family history together with a decline in function. The mean Global Assessment of Functioning score of the group at clinical presentation was 61. Psychopathology on the day of scanning was assessed using the Positive and Negative Symptom Scales (PANSS) (Kay, 1990). The PANSS symptom ratings are presented in Table 1. Subjects were scanned shortly after clinical presentation (mean duration between presentation and MRI scanning = 36.61 days). Two ARMS subjects had received low doses of risperidone and quetiapine at the time of scanning. All the other subjects were naïve to antipsychotic and other forms of psychotropic medication. The subjects will be monitored to determine their long term clinical outcome; this process is ongoing.

#### FMRI task design

The Hayling Sentence Completion Task (Burgess and Shallice, 1996) was adapted for use in a functional MRI experiment. Eighty sentence stems were selected from those created by Bloom and Fischler (1980) and Arcuri et al. (2001). The stems comprised either six or seven words and were selected on the basis of being associated with either a high (> 0.9) or a low (< 0.5) Cloze probability (CP). This is the probability that a particular word will be used to complete a given sentence (Kutas and Hillyard, 1984). The sentences were then assigned to either a response Initiation condition, in which participants were required to complete the sentence with a congruent response (i.e. He posted the letter without a “STAMP”), or a response Suppression condition, in which a non-congruent completion was required (i.e. The boy went to an expensive “GIRAFFE”). This yielded a factorial design, with congruency (Initiation and Suppression) and constraint (low CP and high CP) as factors. The forty sentence stems in each of the congruency conditions were arranged into blocks containing five stems each. Sentence stems were presented visually one at a time. The experimental conditions were contrasted with a control condition which consisted of overt articulation of the word ‘REST’ presented visually every 4 seconds after a fixation cross also lasting for 4 seconds (Word Repetition). None of the participants reported any difficulty in reading the sentences within the allotted presentation time. A detailed description of the task used is provided in Allen et al. (2008).

### Data acquisition

Images were acquired in a 1.5 T scanner (Signa LX – GE, Milwaukee, USA), using a TR of 2 seconds, flip angle of 80°, TE of 40 ms, 64 × 64 pixels, field of view of 200 mm, slice thickness of 7 mm, and interslice gap of 0.7 mm. In order to optimise the nature of the fMRI data for dynamic causal modelling a continuous acquisition sequence was used as opposed to a clustered acquisition sequence. There were no significant differences between control and ARMS subjects for any motion parameters (all translation and rotation parameters *p* > 0.20). A total of 600 image volumes were acquired in two runs (300 Initiation and 300 Suppression), each lasting 10 minutes. Each brain volume consisted of 16 axial slices parallel to the AC–PC line giving whole brain coverage.

### Behavioural data analysis

Response errors in the Initiation condition occurred when participants gave a response that did not complete the preceding sentence stem in an expected or sensible way. Errors in the Suppression condition were defined as any response that completed the sentence in a sensible fashion or had an obvious connection in meaning to the preceding sentence stem. The appropriateness of each completion in the Suppression condition was defined in accordance with the Hayling and Brixton Tests section 5 (Thames Valley Test Company Ltd, 1997). When there was uncertainty as to the accuracy of a response a consensus decision was made between three investigators. Repeated measures ANOVA was used to analyse mean error rates and reaction times.

### FMRI data analysis

Preprocessing and statistical analysis of functional data were performed using SPM2 software (http//www.fil.ion.ucl.ac.uk/spm), running in Matlab 6.5 (Mathworks Inc. Sherbon, MA, USA). All volumes from each subject were realigned using the first as reference and resliced with sinc interpolation. The functional images were spatially normalized (Friston et al., 1995) to a standard MNI-305 template using nonlinear-basis functions. Functional data were spatially smoothed with a 6 mm full width at half maximum isotropic Gaussian kernel to compensate for residual variability in functional anatomy after spatial normalisation and to facilitate application of Gaussian random field theory for adjusted statistical inference.

We performed a standard voxel-wise statistical analysis, using a general linear model, in order to identify regional activations, in each subject independently. To remove low-frequency drifts, the data were high-pass filtered using a set of discrete cosine basis functions with a cutoff period of 128 seconds. The four experimental conditions, i.e. Initiation (High CP), Initiation (Low CP), Suppression (High CP), Suppression (Low CP), and Word Repetition (Control condition), were modelled independently by convolving the onset times (from the onset of the question mark prompting a verbal response) with a canonical haemodynamic response function. Error responses were modelled by a separate regressor to remove them from the analysis. Serial correlations among scans were modelled using an AR(1) model, enabling maximum likelihood estimates of the whitened data. The parameter estimates were calculated for all brain voxels using the general linear model and entered into a second-level random effects analysis. Separate within-group ANOVA were used to examine the main effects of response congruency (Initiation vs. Suppression) for both healthy controls and ARMS subjects. As the within-group main effect for constraint (high vs. low CP) was non-significant in both groups High and Low CP conditions were collapsed and this factor dropped from subsequent analyses. A subsequent between-group 2 × 2 factorial ANOVA was used to examine group effects and group-by-task interactions. Statistical inferences were made at a whole-brain corrected cluster level (*p* < 0.05, with a standard voxel-level threshold of *p* < 0.001).

### Dynamic causal modelling

We used dynamic causal modelling (Friston et al., 2003) as implemented in the SPM5 software. The general goal of DCM is to explain regional effects (as detected by a conventional general linear model) in terms of connectivity and its experimentally induced modulation (cf. (Stephan et al., 2007a). In the present study, the results of the general linear model analysis described below motivated a DCM analysis that focused on explaining activity in left middle temporal gyrus (LMTG), the left middle frontal gyrus (LMFG), and the anterior cingulate (ACC) in terms of task-dependent changes in their connectivity. DCM models show how the neural dynamics are shaped by experimentally controlled manipulations such as stimulus presentation or task instruction. Inputs can elicit responses through direct influences on specific regions (driving inputs) or they can change the strength of coupling among regions (modulatory inputs). DCM also allows the characterization of coupling between regions irrespective of task modulation (endogenous connections).

The estimated underlying neural activity is then used to derive the connectivity parameters, as described elsewhere (Friston et al., 2003).

We ensured comparability across subjects and groups by requiring that the extracted time series met a combination of anatomical and functional criteria (cf. Stephan et al., 2007b). Functionally, the choice of subject-specific coordinates was guided by group maxima (see Fig. 2). In healthy controls, the group maximum in LMFG was [−44, 30, 30] (Suppression > Word Repetition) and in LMTG [−62, −36, −4] (Initiation > Word Repetition). In ARMS subjects, the group maximum in LMFG was [−40, 2, 46] (Suppression > Word Repetition) and in LMTG [−52, −50, −6] (Initiation > Word Repetition). The ACC was located at [6, 12, 26], as defined by the significant simple main effect (ARMS > healthy controls) during Suppression (Fig. 3b). We then chose subject-specific maxima in these regions that were (i) within a radius of 12 mm around the group maxima and (ii) within the same gyrus. Regional time series were extracted as the first eigenvariate of all activated voxels within a 12 mm radius around the subject-specific maxima. We failed to find regions that conformed to these criteria in four subjects (two controls and two ARMS subjects) which were therefore excluded from the DCM analysis.

In a previous study of healthy subjects, we characterised fronto-temporal coupling using a two-region model (with frontal and temporal regions). In the current study, we used the same paradigm, in healthy and ARMS subjects, and found a significant group difference in the anterior cingulate cortex. Therefore, we extended our previous model to include a third (anterior cingulate) region. Our analysis procedure involved optimising this three-area model, using Bayesian model selection (BMS), and then testing for group differences in both its endogenous and bilinear parameter estimates using classical inference. BMS was based upon a novel random effects model that accounts for between-subject heterogeneity in terms of which model best explained their measured data (Stephan et al., 2009a, 2009b); this random effects approach is the method of choice for clinical studies. We optimised our three-area DCM (see below for details) both by considering all subjects together and by treating each group separately. The results were consistent, yielding the same optimal model; to demonstrate this we report the results of model selection for each group separately.

A basic three-area DCM was specified with bidirectional endogenous connection between all regions (LMTG, LMFG, and ACC) and with driving input into LMTG. This base model was then modified systematically to produce fourteen alternative model variants. Specifically, we were interested in the role of the ACC in ARMS subjects and how connections between ACC, LMTG, and LMFG were modulated by task demands (i.e. Suppression and Initiation; modulatory inputs in this group). Fig. 3a shows the three-area Models 1 to 4; an additional 10 models were constructed by combinations of these models (Model 5 = 1 + 2, Model 6 = 1 + 3, Model 7 = 1 + 4, Model 8 = 2 + 3, Model 9 = 2 + 4, Model 10 = 3 + 4, Model 11 = 1 + 2 + 3, Model 12 = 1 + 2 + 4, Model 13 = 1 + 3 + 4, and Model 14 = 2 + 3 + 4).

### Bayesian Model Selection (BMS)

After constructing a series of three-area DCMs in each subject we then compared these models using Bayesian model selection. BMS not only takes into account the relative fit of competing models but also their relative complexity (e.g. number of free parameters, functional form). A detailed explanation of BMS is provided by Penny et al. (2004). To avoid biasing our analysis towards a model found to be optimal in controls subjects, model selection was carried out for both groups independently. The results were consistent, i.e. the same optimal model was found in both groups (see below). We used a novel Bayesian method for model comparison at the group level which deals gracefully with outliers and represents a random effects analysis (Stephan et al., 2009a, 2009b). In short, this method estimates the probability densities of the models themselves, given the measured data across the group. It rests on a variational Bayes approach to estimate the parameters of a Dirichlet distribution approximating the posterior density of the model probabilities; these parameter estimates define a multinomial distribution of how likely it is that a specific model generated the data from a specific subject. In particular, this method allows one to quantify so-called exceedance probabilities, i.e. the probability that a given model is more likely than any other model tested.

Having identified the best three-region model we then tested for consistent group differences in the effective connectivity or coupling coefficients using ANOVA; with connection type (including endogenous connections and condition-specific bilinear modulations) as within-subject factor and group as a between-subject factor. Post-hoc analysis used independent sample *t*-tests to compare connectivity parameter estimates between ARMS and healthy control subjects (Bonferroni correction for multiple comparisons was applied).

## Results

### Participants

Healthy controls and ARMS subjects were matched for age, estimated premorbid IQ, gender, and ethnicity (Table 1).

### Behavioural performance

**Fig. 1:**
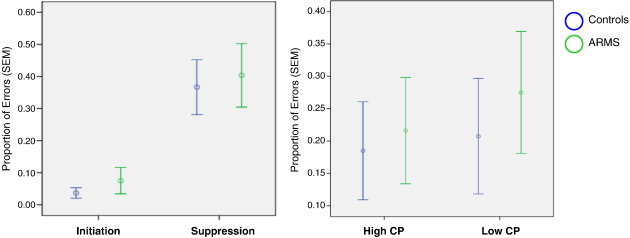
Proportion of errors (SEM) for response Initiation and Suppression, High and Low CP by Group.

The proportion of errors during the response Suppression and Initiation conditions are shown in Figs. 1a and b. There was a significant main effect of congruency (*F* = 139.94; *df* = 1, 28; *p* < 0.001), with both groups making more errors during response Suppression than Initiation. There was also a significant main effect of constraint (*F* = 6.77; *df* = 1, 28; *p* < 0.01): both groups made more errors when completing Low than High CP sentence stems. There were no significant interactions between congruency and group (*F* = 0.007; *df* = 1, 28; *p* = 0.93), between constraint and group (*F* = 0.83; *df* = 1, 28; *p* = 0.35), between or congruency, constraint, and group (*F* = 0.45; *df* = 1, 28; *p* = 0.51). Although reaction times were relatively slower for response Suppression and Low CP trials, neither was there a significant main effect of congruency or constraint on reaction time nor were there significant interactions between congruency or constraint with group (all effects *p* > 0.40).

### FMRI

#### Healthy controls

Although the fMRI results for the healthy control subjects have been reported before (Allen et al., 2008), we briefly summarise them at this point to enable direct comparison with the results for ARMS subjects described below. Controls showed activation during response Initiation *relative* to Word Repetition in the left superior frontal gyrus, ventrolateral inferior frontal gyrus, middle frontal gyrus, middle temporal gyrus, cuneus, and in the superior temporal pole bilaterally (Fig. 1b). During response Suppression, compared to word repetition, there was activation in the same areas, with additional activation in the left precentral gyrus (Fig. 1c). When response Suppression compared to Initiation directly, there was activation in the left middle temporal gyrus, the left orbital gyrus, and the precuneus bilaterally (Fig. 1d). There were no areas that showed greater activation during response Initiation than response Suppression. The main effect for response constraint (Low vs. High CP conditions) was non-significant. Exact coordinates and statistics of these results can be found in Allen et al. (2008).

#### ARMS subjects

During response Initiation, ARMS subjects showed activation relative to word repetition in the left insula and the left superior, middle, and inferior temporal gyri (Fig. 1e; Table 2). During response Suppression, compared to word repetition, activation was seen in the left inferior frontal gyrus extending into the inferior precentral sulcus, the left middle frontal gyrus, insula, middle and superior temporal gyri, and in the medial superior frontal gyrus bilaterally (Fig. 1f). No areas showed a significant main effect for response congruency or constraint.

#### ARMS vs. healthy controls

**Fig. 2:**
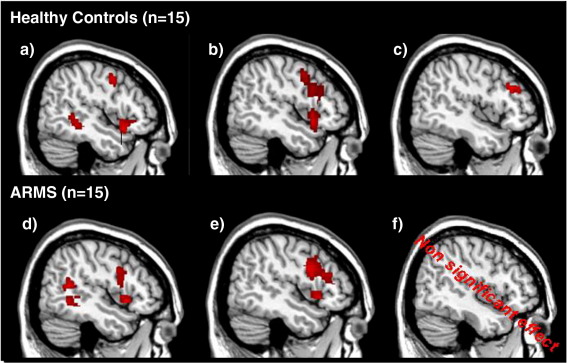
Statistical parametric maps during (a) response Initiation > Word Repetition in controls, (b) Suppression > Word Repetition in controls, (c) Suppression > Initiation in controls, (d) Initiation > Word Repetition in ARMS, (e) Suppression > Word Repetition in ARMS, and (f) Suppression > Initiation in ARMS . All activations are reported at a whole-brain corrected cluster threshold of *p* < 0.05 (with a standard voxel-level cutoff of *p* < 0.001). Coordinates for activated regions in ARMS are presented in Table 2. Coordinates of activations in healthy control subjects are reported in Allen et al (2008).

Across both the response Initiation and Suppression conditions, ARMS subjects showed more activation than healthy controls in the right caudate and the ACC bilaterally (main effect of group; Fig. 2a; Table 3). There were no areas showing greater activation for healthy controls than in ARMS subjects. Additionally, there was a significant group-by-task interaction in the ACC. Post-hoc two-sample *t*-tests revealed that during response Suppression, but not Initiation, ARMS showed significantly increased activation compared to controls in this region (Fig. 2b; Table 3).

## DCM analysis

**Fig. 3:**
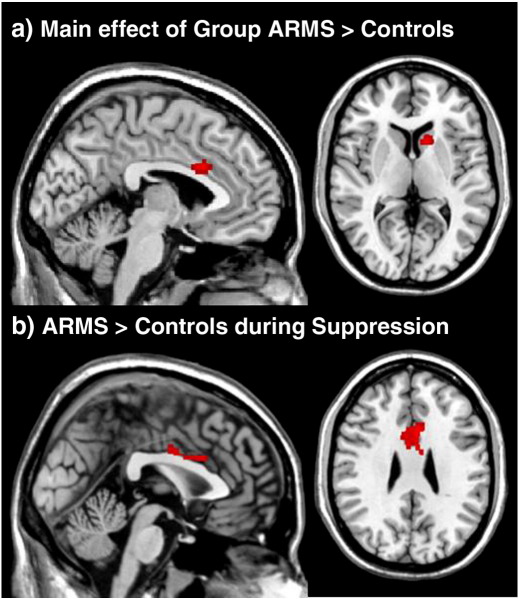
Statistical parametric maps showing (a) the main effect of group across both response Initiation and Suppression and (b) simple main effect of group during response Suppression in the ACC. All activations are reported at a whole-brain corrected cluster threshold of *p* < 0.05 (with a standard voxel-level cutoff of *p* < 0.001). Coordinates for activated regions are presented in Table 3.

**Fig. 4:**
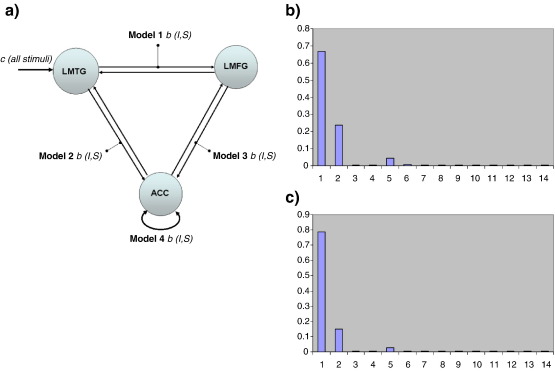
(a) Competing three-area DCMs of effective connectivity constructed with bidirectional connections to/from ACC, LMTG, LMFG. Models 1 to 4 specify different locations for modulatory inputs (b); an additional 10 models were constructed by combinations of these models (Model 5 = 1 + 2, Model 6 = 1 + 3, Model 7 = 1 + 4, Model 8 = 2 + 3, Model 9 = 2 + 4, Model 10 = 3 + 4, Model 11 = 1 + 2 + 3, Model 12 = 1 + 2 + 4, Model 13 = 1 + 3 + 4 and Model 14 = 2 + 3 + 4). (b) Exceedance probabilities for three-area Models 1–14 in controls. (c) Exceedance probabilities for three-area Models 1–14 in ARMS subjects. *X*-axis = model number, *Y*-axis = exceedance probability.

Following construction and inversion of 14 alternative DCMs per subject (see Materials and methods section and Fig. 3a), random effects Bayesian model selection (Stephan et al., 2009a, 2009b) showed that in both groups Model 1 clearly outperformed all other models, with an exceedance probability of 67% in controls (Fig. 4b) and of 79% in ARMS subjects (Fig. 4c). The optimal three-area Model 1 contained reciprocal endogenous connections from the ACC to both temporal and frontal areas with task-dependent modulation of the forward connection between the MTG and MFG only. Both endogenous and modulatory parameters were then submitted to a subsequent statistical group analysis. Repeated measures ANOVA revealed a significant connection-by-group interaction (*F* = 2.27; *df* = 7, 128; *p* = 0.03) indicating an overall group difference in network architecture. Post-hoc *t*-tests revealed that task-dependent modulations (bilinear terms) of the LMTG to LMFG connection did not differ significantly between healthy control and ARMS subjects (Table 4). Analysis of endogenous connectivity parameters revealed that LMTG to ACC, LMTG to MFG, and ACC to LMFG connectivity was greater in ARMS subjects relative to controls. After Bonferroni correction for multiple comparisons only the group difference in LMTG to ACC connectivity remained significant (Table 4).

## Discussion

The aim of the present study was to examine fronto-temporal effective connectivity in the ARMS. Functional MRI was used to measure changes in cerebral activation during the verbal generation of semantically congruent (Initiation) and incongruent (Suppression) responses to a pre-selected sentence. As only two ARMS participants had been exposed to antipsychotic medication, the effects of treatment on group differences in activation are likely to have been minimised.

Both healthy controls and ARMS subjects completed response Initiation trials with a high degree of accuracy, but as would be expected, error rates increased significantly during response Suppression and when the sentences had a Low CP. Neither were there significant group differences for errors in any of the response conditions neither were there any differences for reaction times. It is worth noting that the study design was not powered to identify behavioural differences. Indeed, the presence of significant effects on activation but not on task performance is a common finding in neuroimaging studies as functional neuroimaging techniques, which detect changes at the physiological level, are often intrinsically more sensitive than behavioural measures (Wilkinson and Halligan, 2004).

In both healthy control and ARMS subjects, response Initiation and Suppression were associated with activation in a predominantly left-sided network of prefrontal and lateral temporal regions, consistent with previous imaging studies of the HSCT (Collette et al., 2001; Nathaniel-James et al., 1997; Nathaniel-James and Frith, 2002). In healthy controls, response Suppression was associated with more activation in the LMFG than response Initiation. This may reflect the increased cognitive demands of this condition, specifically in terms of the inhibition of a pre-potent response and strategy formation (de Zubicaray et al., 2000). In contrast, in the ARMS group, there were no regions that were significantly more activated during response Suppression than Initiation. Direct comparison of activation in the ARMS and control groups revealed that the ARMS group showed greater engagement of the anterior cingulate gyrus and caudate, but, contrary to our hypothesis, there were no differences in fronto-temporal activation. Although altered activation in frontal and temporal regions has been widely reported in schizophrenia (Ragland et al., 2007; Weiss and Heckers, 2001), a previous study using the HSCT in another high risk group also failed to identify differential activation in fronto-temporal regions (Whalley et al., 2004). The group differences were largely driven by greater engagement of the ACC during response suppression in ARMS subjects than controls. In the present study increased cingulate activation in ARMS subjects during response Suppression may facilitate the selection of incongruent responses. It is proposed that during normal cognition the anterior cingulate serves a specific evaluation function, detecting response competition or conflict and indicating the need to implement strategic processes to reduce conflict and maintain performance (Yücel et al., 2003; Carter et al., 1999, 2000).

### Dynamic causal modelling

To investigate the effective fronto-temporal connectivity in ARMS subjects, and the role of differential cingulate activation in this, we constructed a series of three-area DCMs. Given the task by group interaction in the ACC, we investigated the possibility that increased ACC activity could represent a compensatory mechanism in ARMS, with the ACC influencing activity in temporal and frontal areas and thereby also influencing their connectivity. Using BMS we systematically compared 14 competing models of temporal, frontal, and cingulate integration during response Initiation and Suppression in both groups independently. A model in which driving inputs entered via the LMTG and in which LMTG to MFG connectivity was allowed to vary between Suppression and Initiation in the presence of bidirectional connections with the ACC (Model 1) was found to be optimal for explaining the measured fMRI responses in both ARMS subjects and healthy controls. In this model, across all subjects, the modulatory effects on temporal to frontal connectivity were significantly greater for response Suppression compared to Initiation. However, contrary to our prediction the modulatory parameters for response Initiation and Suppression did not differ significantly between groups, indicating intact effective connectivity between temporal and frontal regions in ARMS subjects. Subsequent analysis of endogenous connections did, however, reveal significant group differences in connectional architecture. After correction for multiple comparisons, ARMS subjects showed greater effective connectivity from the LMTG to ACC relative to healthy controls. ARMS also had greater effective connectivity from the ACC to the LMFG, albeit at an uncorrected threshold (*p* < 0.05). Our finding of relatively increased activation of the ACC *and* increased effective connectivity between this region and temporal and frontal areas in the ARMS would be compatible with the engagement of a mechanism that compensated for a fronto-temporal system which was faulty, but had not yet (as in schizophrenia) failed. The cingulate cortex has strong reciprocal connections with prefrontal (Petrides and Pandya, 1988) and superior temporal cortex (Pandya et al., 1981). Interestingly, in a study by Fletcher et al. (1999), cingulate modulation of fronto-temporal connectivity was seen in controls but not in patients with schizophrenia. This is in line with reports that patients with schizophrenia tend to exhibit *reduced* activation of the anterior cingulate cortex compared to controls during a wide range of cognitive tasks (van Veen and Carter, 2002), particularly tasks that entail word generation (Dolan et al., 1995) and selecting between competing responses (Carter et al., 1997). Thus, the increase in cingulate activity found in ARMS subjects may allow for the normal patterns of frontal and temporal activation and normal task-dependent modulation of fronto-temporal connectivity we observed in the ARMS.

However, in post-hoc analyses of endogenous connection parameters we also found increased endogenous (fixed or non-modulated) effective connectivity from LMTG to LMFG in ARMS subjects relative to healthy controls (albeit at an uncorrected statistical threshold only). It is unclear why ARMS subjects may exhibit greater effective connectivity between these regions; this could also be a consequence of the increased coupling between the ACC and both frontal and temporal regions.

The study has some limitations. First, we were unable to compare ARMS subjects based on their clinical outcome. We are currently in the process of identifying the ARMS subjects who have subsequently made the transition to first episode psychosis. Second, it should be noted that our model only tested for linear influences of ACC on LMTG and LMFG but did not test whether ACC activity exerted a non-linear influence on temporal-frontal connections (cf. Stephan et al. 2008); this will be the subject of future work. Moreover, increased cingulate activation may be compensating for inefficiency in multiple brain areas and multiple functional contexts not just fronto-temporal regions during language processing. The reasons for such inefficiency, however, remain unclear and are beyond the scope of this study. Finally, our study was not designed to address the relationship between disturbances in effective and structural connectivity; this will be addressed by future investigations.

In conclusion, although we found no evidence of altered fronto-temporal activation or task-dependent modulation of connectivity in the ARMS, the maintenance of fronto-temporal integration in the ARMS may depend on a compensatory activation in the cingulate cortex. Further work is required to examine the extent to which the modulatory influence of the cingulate on fronto-temporal integration may change as high risk subjects proceed to develop a psychotic disorde.

## Figures and Tables

**Table 1:** Mean and standard deviations for demographic characteristics and symptom ratings.

	Healthy controls (*n* = 15)	ARMS (*n* = 15)	Analysis
Age (years)	25.75 (4.95)	26.85 (4.95)	*t* = 1.23; *p* = 0. 21
Gender	8M: 7F	9M: 6F	*χ*^2^ = 0.05; *p* = 0.81
WRAT estimated premorbid IQ	105 (15.47)	103 (15.66)	*t* = 0.36; *p* = 0.75
Years of education	14.89 (3.11)	13.76 (2.87)	*t* = 0.1.56; *p* = 0.18
Symptoms			
PANSS total		47 (13)	
PANSS positive		11 (4)	
PANSS negative		11 (4)	
PANSS general		24 (7)	

**Table 2:** Coordinates and *Z* scores (cluster level corrected for comparisons across the whole brain, *p* < 0.05, with a voxel-level threshold of *p* < 0.001) for cerebral areas activated in ARMS subjects.

Region	*x*	*y*	*z*	*Z* score
*Initiation > Word Repetition*				
L superior temporal sulcus	−50	−48	14	5.56
L middle temporal gyrus	−58	−44	−6	5.26
L middle temporal gyrus	−52	−50	−6	4.94
L insula	−48	16	−4	4.93
	−38	24	−4	4.73
L inferior frontal gyrus	−58	18	26	4.46

*Suppression > Word Repetition*				
L inferior frontal gyrus	−58	18	26	5.57
	−48	8	20	4.09
L inferior precentral sulcus	−46	8	28	5.16
L superior frontal gyrus	−2	16	52	5.44
R superior frontal gyrus	10	16	46	3.37
L cingulate gyrus	−2	28	32	3.37
L inferior frontal gyrus (pars orbitalis)	−46	18	−4	4.48
L superior temporal gyrus (planum polare)	−52	10	−2	4.56
L insula	−36	24	−4	4.22
L middle temporal gyrus	−58	−42	−8	4.29
	−58	−38	0	4.05
L middle frontal gyrus	−48	2	46	3.98

*Initiation > Suppression and Suppression > . Initiation*				
No significant effects				

**Table 3:** MNI coordinates and *Z* scores (cluster level corrected for comparisons across the whole brain, *p* < 0.05, with a voxel-level threshold of *p* < 0.001) for cerebral areas showing a main effect of group.

Region	*x*	*y*	*z*	*Z* score
Main effect of group				
*Healthy controls > ARMS*				
No Significant effect				
				
*ARMS > Healthy healthy controls*				
R caudate	10	14	8	4.43
R cingulate gyrus	6	18	28	4.20
L cingulate gyrus	−6	14	26	3.94
Group × task interaction				
R cingulate gyrus	6	18	26	4.15
L cingulate gyrus	−4	14	30	4.05
				
*Post-hoc two-sample t t-tests*				
Initiation condition				
No significant effect				
Suppression condition				
				
*ARMS > Healthy healthy controls*				
R cingulate gyrus	6	12	26	4.10
L cingulate gyrus	−4	4	30	3.67
				
*Healthy controls > ARMS*				
No significant effect				

**Table 4:** Mean (standard deviations) DCM modulatory (bilinear terms) and endogenous parameter estimates for all connections in healthy controls and ARMS subjects.

Connection type	Healthy controls (*n* = 13)	ARMS (*n* = 13)	*t*	*P*
*Modulatory parameters*				
MTG → MFG Initiation	−0.09 (0.37)	0.043 (0.26)	−1.12	0.28
MTG → MFG Suppression	0.02 (0.28)	0.20 (0.30)	−1.54	0.13
				
*Endogenous parameters*				
MTG → MFG	−0.018 (0.38)	0.35 (0.36)	−2.5	0.02⁎
MTG → ACC	0.01 (0.18)	0.32 (0.27)	−4.06	< 0.01⁎⁎
MFG → MTG	0.20 (0.41)	0.021 (0.47)	1.02	0.31
MFG → ACC	0.05 (0.22)	0.15 (0.23)	−1.17	0.25
ACC → MTG	0.01 (0.16)	0.01 (0.27)	−0.01	0.99
ACC → MFG	0.01 (0.13)	0.20 (0.27)	−2.21	0.04⁎

⁎ Group difference significant a *p* < 0.05 uncorrected for multiple comparisons.

⁎⁎ Group difference survives Bonferroni correction for multiple comparisons.
